# Prevalence and Epidemiological Characteristics of *Mycoplasma synoviae* Infection in Chickens in Mainland China

**DOI:** 10.3390/ani16121893

**Published:** 2026-06-18

**Authors:** Xinyuan Liu, Huiling Zhang, Zihan Huang, Lu Wang, Hongyu Zhou, Tangjie Zhang

**Affiliations:** 1 Institute of Comparative Medicine, College of Veterinary Medicine, Yangzhou University, Yangzhou 225009, China; 18795961172@163.com (X.L.); 19291919820@163.com (Z.H.); 18912084046@163.com (L.W.); zhou2096177403@foxmail.com (H.Z.); 2 Jiangsu Co-Innovation Center for Prevention and Control of Important Animal Infectious Diseases and Zoonoses, Yangzhou 225009, China; 3 Independent Researcher, New York, NY 11355, USA; huz21@pitt.edu

**Keywords:** *Mycoplasma synoviae*, meta-analysis, seroprevalence, molecular detection prevalence, age groups, breeders, mainland China

## Abstract

*Mycoplasma synoviae* (MS) poses a continuous threat to the poultry industry in Mainland China, yet a comprehensive nationwide assessment remains lacking. This systematic review and meta-analysis quantified the pooled prevalence of MS and evaluated key epidemiological characteristics and sources of heterogeneity. Peer-reviewed studies were retrieved from multiple databases, and random-effects models were utilized to estimate and compare pooled seroprevalence and molecular detection prevalence. The results revealed a heavy MS infection burden characterized by a distinct diagnostic disparity: a high serological prevalence co-existed with a moderate molecular detection prevalence, reflecting widespread past exposure, chronic carrying, or vaccination. Geographically, the Northwest region exhibited the highest seroprevalence (61.8%), though inter-regional differences were not statistically significant (*p* = 0.152). Among production types, breeder flocks maintained a relatively high pooled prevalence of 69.6% (95% CI: 40.5–92.0%), although differences among production types were not statistically significant. Subgroup analysis demonstrated a statistically significant difference among age groups; however, the available data did not support definitive conclusions regarding age-specific risk patterns. Breeder flocks exhibited a relatively higher pooled prevalence than layers and broilers, suggesting a potential epidemiological role that warrants further investigation. In conclusion, MS appears to be widely distributed in chickens in mainland China. Differences between the serological and molecular detection results highlight the complexity of interpreting MS prevalence estimates. Given the substantial heterogeneity among studies, the findings should be interpreted cautiously. Future studies incorporating standardized surveillance and detailed vaccination histories are needed to better understand MS epidemiology and support evidence-based control strategies.

## Simple Summary

*Mycoplasma synoviae* (MS) is a major pathogen causing significant economic losses in the poultry industry worldwide. This study conducted a systematic review and meta-analysis to evaluate MS prevalence in chicken flocks across Mainland China. Our findings reveal a widespread MS circulation characterized by a high antibody detection rate (seroprevalence) but a moderate molecular detection prevalence, indicating that many chickens are in states of past exposure, chronic carrying, or vaccination. Geographically, the Northwest region and breeder flocks showed relatively higher prevalence estimates, although not all subgroup differences were statistically significant. MS prevalence varied significantly among different age groups. Overall, these findings provide updated epidemiological evidence regarding the distribution of MS in chicken populations in mainland China and highlight the need for continued surveillance and further epidemiological investigations.

## 1. Introduction

Mycoplasma synoviae (MS) is one of the most economically damaging pathogens in the global poultry industry. It infects chickens to cause infectious synovitis, airsacculitis, and subclinical infections, frequently accompanied by a severe drop in egg production and eggshell apex abnormality (EAA), thereby leading to substantial economic losses [1,2]. In recent years, driven by the continuous intensification of poultry production and the widespread adoption of multi-age rearing systems, the prevalence patterns of MS have exhibited substantial variation across various farming conditions, with transmission risks escalating continuously [3,4]. Furthermore, MS can spread horizontally within flocks via direct contact and vertically through breeding eggs, which significantly exacerbates the complexity of disease prevention and eradication [3].

Globally, MS is widely distributed across major poultry-producing regions in Asia, Europe, the Americas, and Africa, though the reported prevalence varies markedly among countries and regions [5,6,7]. Epidemiological investigations in China have similarly demonstrated that MS infection is widespread and severe in mainland China. Serological surveys revealed that between 2010 and 2015, the MS antibody positivity rate among chicken flocks across 21 provinces in China reached 41.19%, with striking regional variations ranging from 5.10% to 100% [8]. Another large-scale epidemiological survey reported that more than 9000 chicken flocks across 16 provinces were affected by MS during 2010–2015, with a breeder embryo infection rate of 16.29%, highlighting the critical role of vertical transmission in its epidemic dynamics [1]. Recent studies have further indicated that the MS molecular detection prevalence in suspected clinical samples from central China reached as high as 55.48% during 2021–2023, coupled with the dominance of specific genotypes [9]. Concurrently, a multi-center study in 2024 across 15 provinces reported an MS prevalence of approximately 14% in mainland China, accompanied by high genetic diversity [10].

However, the existing epidemiological findings exhibit profound heterogeneity, and a systematic synthesis of these scattered data remains lacking. A major driver of the discrepancy in prevalence estimates, apart from temporal and spatial shifts, lies in the variations in sampling strategies (e.g., “random populations” in active surveillance versus “suspected cases” in passive diagnosis), diagnostic methods (e.g., serological assays versus molecular tools), and specific host characteristics [9,11,12]. Consequently, the direct comparability between individual studies is limited, hindering the establishment of a comprehensive, nationwide baseline of MS prevalence.

Beyond diagnostic and sampling artifacts, the actual infection dynamics of MS are driven by multifaceted epidemiological factors. These encompass host-specific traits (such as age, breed, and immune status) as well as environmental and management variables (including stocking density, multi-age housing, and biosecurity protocols) [3,4]. Previous literature indicates that young chicks aged 3–7 weeks are highly susceptible to MS, representing a critical window for horizontal transmission [3], whereas infection or insufficient maternal antibody levels in breeder flocks directly elevate the risk of embryonic infection and subsequent EAA-related performance losses [2]. Nevertheless, current insights into these epidemiological factors and prevalence patterns are predominantly derived from single-region or localized studies, and the consistency and generalizability of their conclusions warrant rigorous verification on a broader national scale.

To date, although numerous epidemiological investigations on MS have been conducted in China, there is an absence of a systematic review and meta-analysis providing a quantitative synthesis of the epidemiological characteristics and sources of epidemiological variation in MS in mainland China [13]. Therefore, it is highly necessary to comprehensively aggregate historical data using a meta-analytic approach to clarify the overall prevalence and to explore factors potentially associated with variations in prevalence estimates.

To resolve these inconsistencies, we pooled and evaluated eligible cross-sectional data published over the past decade to establish a reliable baseline for MS prevalence across mainland China. Furthermore, subgroup analyses were performed to dissect the impacts of geographical region, sampling period, diagnostic method, age, production type, and sampling strategy on the pooled prevalence estimates. The findings of this study provide a comprehensive synthesis of the currently available evidence on MS prevalence in mainland China and may contribute to future surveillance, epidemiological investigations, and evidence-based disease control strategies.

## 2. Materials and Methods

### 2.1. Literature Search Strategy and Criteria for the Selection of Studies

This systematic review and meta-analysis were conducted and reported in strict accordance with the Preferred Reporting Items for Systematic Reviews and Meta-Analyses (PRISMA) guidelines. A comprehensive and systematic literature search was performed across three Chinese databases—China National Knowledge Infrastructure (CNKI), Wanfang, and VIP—and two international databases—PubMed and Web of Science. The search covered studies published from January 2014 to March 2026. A combination of controlled vocabulary (such as MeSH terms) and free-text terms in both English and Chinese was utilized. The core search string was defined as follows:

(“Mycoplasma synoviae” OR “MS”) AND (“prevalence” OR “infection rate” OR “seroprevalence”) AND (“chicken” OR “poultry”) AND (“China” OR “Chinese mainland”)

Additionally, the reference lists of all retrieved articles and relevant reviews were manually screened (“backward citation tracking”) to capture potentially eligible studies that might have been missed during the electronic database search.

The literature selection process involved an initial screening of titles and abstracts to eliminate obviously irrelevant literature, followed by a rigorous full-text review. Studies were eligible for inclusion if they met the following criteria: (1) geographical location: The study was conducted in mainland China and reported original, individual-level data (i.e., the number of positive cases and the total sample size. In this study, “mainland China” refers to provincial-level administrative regions of the People’s Republic of China excluding Taiwan, Hong Kong, and Macau; (2) study design: Cross-sectional investigations or routine epidemiological surveillance; (3) diagnostic methods: Standardized serological tests (e.g., enzyme-linked immunosorbent assay [ELISA], serum platelet agglutination [SPA], hemagglutination inhibition [HI]) or molecular detection methods (e.g., polymerase chain reaction [PCR], isolation and culture); (4) data availability: Clarified the prevalence of MS or provided sufficient data to directly extract or calculate the number of positive samples and the total examined samples, for each eligible study, the numbers of positive birds and total examined birds were extracted. The meta-analysis was therefore conducted primarily at the individual bird level rather than the flock level whenever such information was available. (5) sampling strategy: Samples were obtained via random sampling or routine active monitoring, rather than targeted selection based on clinical symptoms.

Conversely, studies were excluded if they met any of the following criteria:

Investigations focusing exclusively on clinically suspected cases or outbreak investigations; (1) studies with incomplete data where the exact sample size or positive counts could not be retrieved; (2) duplicate publications, review articles, conference abstracts, or editorials; (3) studies with a total sample size of fewer than 200 chickens; (4) low-quality studies as determined by the methodological quality assessment.

The entire screening, eligibility assessment, and inclusion procedure were independently executed by two investigators. Any discrepancies or disagreements during the selection process were resolved through comprehensive discussion and consensus; if an agreement could not be reached, a third senior reviewer was consulted to make the final determination.

### 2.2. Data Extraction and Quality Assessment

A standardized, pre-piloted Excel spreadsheet was used to extract data from the eligible studies. Data extraction was conducted independently by two researchers and cross-checked to ensure consistency. For each included study, the following variables were extracted: first author, publication year, sampling period, geographical region, total sample size, number of positive cases, production type (e.g., broilers, layers, breeders), age of chickens, and diagnostic methodology (antibody vs. antigen/nucleic acid detection). For age-based subgroup analyses, studies were categorized as <90 days or ≥90 days because this threshold was the most consistently reported age classification among the included studies and provided sufficient data for quantitative synthesis.

The methodological quality of the cross-sectional studies was quantitatively assessed using the Joanna Briggs Institute (JBI) Critical Appraisal Checklist for Studies Reporting Prevalence Data (2020 version) [14]. This tool encompasses eight specific domains: (1) Was the sampling frame appropriate to address the target population? (2) Were study participants recruited in an appropriate way? (3) Was the sample size adequate? (4) Were the study subjects and setting described in detail? (5) Was data analysis conducted with sufficient coverage of the identified sample? (6) Were valid and reliable methods used for the measurement of the condition? (7) Was there appropriate statistical analysis? (8) Was the response rate adequate, and if not, was the low response rate managed appropriately? Each item was scored as “Yes,” “No,” or “Unclear”.

### 2.3. Statistical Analysis

All data processing, pooling, and quantitative analyses were executed using Stata software (version 16.0; StataCorp, College Station, TX, USA). To stabilize variances and overcome the issues of non-normality and out-of-bounds confidence intervals associated with proportions close to 0 or 1, the Freeman–Tukey double-arcsine transformation was applied to the raw prevalence data before pooling [15,16]. The final pooled prevalence estimates and their corresponding 95% confidence intervals (95% CIs) were back-transformed to the original proportions for presentation.

Given the fundamental operational differences between diagnostic modalities, studies were strictly stratified into antibody-detection groups (ELISA, SPA, HI) and antigen/nucleic acid-detection groups (PCR) for separate analysis. Heterogeneity among the included studies was statistically evaluated using Cochran’s Q test and quantified by the I^2^ statistic. Heterogeneity was considered low if *p* ≥ 0.10 and I^2^ < 50%, in which case a fixed-effects model was utilized. Conversely, if *p* < 0.10 or I^2^ > 50%, severe heterogeneity was indicated, and a random-effects model was preferred for calculating the pooled prevalence.

To explore potential sources of heterogeneity, systematic subgroup analyses and meta-regression analyses were performed based on pre-specified covariates, including geographical region, sampling year, season, age group, production type, and sampling strategy. These analyses were conducted to investigate factors contributing to between-study variability rather than to identify causal risk factors.

### 2.4. Assessment of Publication Bias and Sensitivity Analysis

Publication bias was visually inspected using funnel plots and further quantified statistically using Egger’s linear regression test, with a *p*-value < 0.05 indicating statistically significant asymmetry and potential publication bias [17]. To evaluate the stability and robustness of the pooled estimates, a sensitivity analysis was conducted using a leave-one-out approach, wherein the meta-analysis model was iteratively re-estimated by sequentially excluding one individual study at a time to assess whether any single dataset exerted an undue influence on the overall results.

## 3. Results

### 3.1. Literature Search and Selection Outcomes

**Figure 1:**
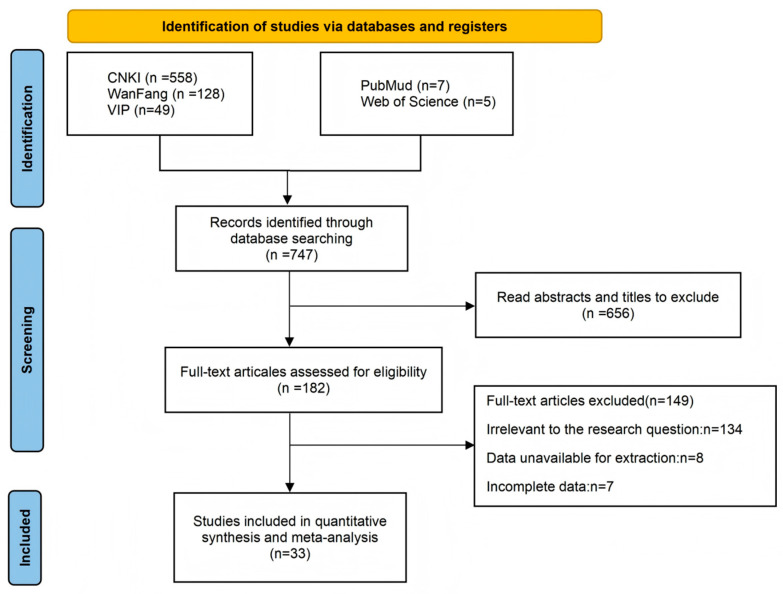
Flow diagram of reference screening.

A flow diagram outlining the systematic literature selection process is illustrated in Figure 1. A total of 747 records were initially retrieved from the five electronic databases within the designated timeframe (1 January 2014 to 31 March 2026). After removing duplicate publications and conducting a preliminary screening based on titles and abstracts, 565 records were excluded as they failed to meet the baseline eligibility criteria. The remaining 182 articles were subjected to a rigorous full-text evaluation. Based on the pre-specified inclusion and exclusion criteria, 149 articles were further excluded for the following reasons: 134 were irrelevant to the specific prevalence of MS in chickens, 8 contained data that could not be mathematically extracted, and 7 lacked complete reporting of essential epidemiological information. Ultimately, 33 eligible studies were included in this meta-analysis.

Notably, although the literature search spanned from January 2014 to March 2026, the actual timeline of field data collection across the included studies was concentrated between 2010 and 2025, with their respective publication dates ranging from 2015 to 2026 [1,8,18,19,20,21,22,23,24,25,26,27,28,29,30,31,32,33,34,35,36,37,38,39,40,41,42,43,44,45,46,47,48]. A cumulative sample size of 93,638 chickens was encompassed in this analysis. The sample sizes of individual studies exhibited substantial variation, ranging from 237 to 44,395 subjects. The comprehensive baseline characteristics of the 33 eligible studies are summarized in Table 1, while the types of chicken production are presented in Supplementary Materials Data S1.

### 3.2. Methodological Quality Assessment of Included Studies

The methodological quality of the 33 included studies was assessed using the standardized JBI Critical Appraisal Checklist. Overall, the included literature demonstrated good methodological rigor and consistency. Among the 33 studies, 11 (33.3%) fulfilled all eight JBI criteria, 14 (42.4%) fulfilled seven of eight criteria, 7 (21.2%) fulfilled six of eight criteria, and only 1 (3.0%) fulfilled five of eight criteria. These findings indicate that the included studies were generally of moderate to high quality, meeting the predefined methodological criteria and providing an acceptable basis for the subsequent meta-analysis. The detailed domain-specific scores and final quality tier assignments for each study are presented in Supplementary Materials Data S2.

### 3.3. Stratified Synthesis and Heterogeneity Analysis

The epidemiological assessment of MS prevalence in chickens primarily relies on two distinct diagnostic paradigms: serological assays (e.g., ELISA, SPA, HI) and etiological/molecular detection methods (e.g., PCR, bacterial isolation). Because these diagnostic approaches target entirely different biological markers (circulating host antibodies vs. actively shedding nucleic acids or live bacteria) and differ fundamentally in their diagnostic windows, sensitivity, and specificity, their corresponding prevalence estimates possess an inherent lack of direct comparability. To minimize the confounding effect of this profound methodological heterogeneity, a strict top-level stratification based on the diagnostic category was implemented prior to effect size pooling.

Among the 33 eligible papers, 19 exclusively reported serological data, 7 exclusively reported molecular detection data, and 7 investigated both modalities simultaneously.

**Figure 2:**
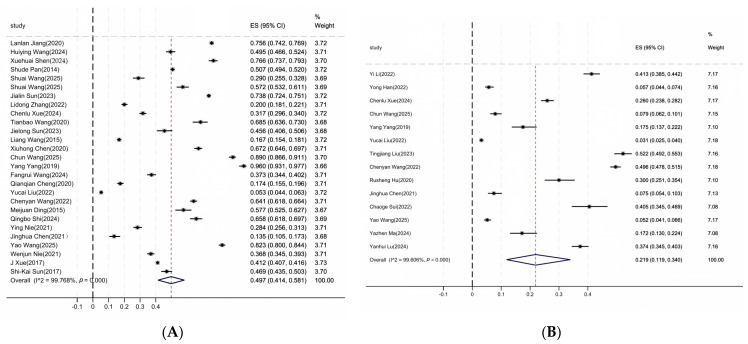
Pooled seroprevalence and molecular detection prevalence estimates of MS using random-effects models (**A**): Forest plot of serological detection. (**B**): Forest plot of molecular detection. The dot represents the prevalence and the horizontal line represents the 95% confidence interval, which corresponds to effect size (ES); the diamond represents the summarized effect [1,8,18,19,20,21,22,23,24,25,26,27,28,29,30,31,32,33,34,35,36,37,38,39,40,41,42,43,44,45,46,47,48].

Serological Prevalence: Based on 27 independent datasets extracted from 26 serological studies, Cochrane’s test revealed extreme and significant inter-study heterogeneity (I^2^ = 99.77%, *p* < 0.001). Consequently, a random-effects model was employed for effect size pooling. The overall pooled seroprevalence of MS among chickens in mainland China was 49.7% (95% CI: 41.4–58.1%), as shown in Figure 2A, suggesting that MS is commonly detected in poultry populations. However, substantial between-study heterogeneity was observed, and therefore this estimate should be interpreted cautiously.

Molecular detection prevalence: Analysis of 14 independent datasets from 14 molecular studies likewise confirmed a high level of heterogeneity (I^2^ = 99.61%, *p* < 0.001). Utilizing a random-effects model, the pooled etiological/molecular detection prevalence of MS was determined to be 21.9% (95% CI: 11.9–34.0%), with the corresponding forest plot illustrated in Figure 2B.

### 3.4. Subgroup Analysis and Exploration of Heterogeneity Sources

Given that substantial residual heterogeneity persisted within both the serological and nucleic acid detection arms (I^2^ > 99%), systematic subgroup analyses and meta-regression were undertaken within each stratified cohort to elucidate potential sources of heterogeneity and to quantitatively appraise the impact of crucial epidemiological covariates. These investigations were executed across five predefined dimensions: sampling/publication year, geographical distribution, production type, age group, and sampling season.

At the macro level, the estimated pooled seroprevalence of 49.7% (95% CI: 41.4–58.1%) was dramatically and significantly higher than the estimated pooled nucleic acid detection prevalence of 21.9% (95% CI: 11.9–34.0%), revealing a highly significant divergence between the two diagnostic modalities (*p* < 0.001; Table 2).

The comprehensive, granular outcomes of the subgroup analyses for both diagnostic paradigms—including stratified pooled prevalence estimates, intra-group I^2^ statistics, and inter-group comparative *p*-values—are systematically summarized in Table 3.

**Figure 3:**
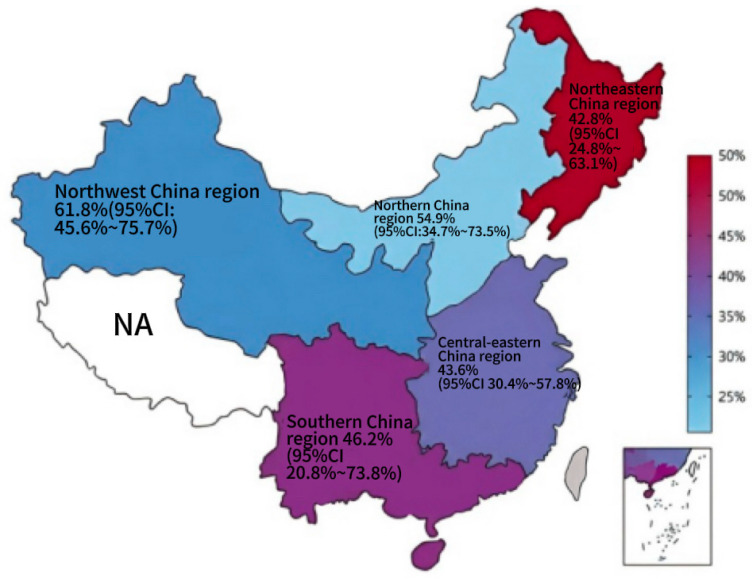
Subgroup analysis of MS by geographical regions in mainland China. NA: not available epidemiological data.

Based on serological testing methods, temporal subgroup analysis revealed an increasing trend in the weighted pooled prevalence of MS, rising from 38.2% (95% CI: 26.4–50.7%) in 2010–2014 to 47.6% (95% CI: 30.4–65.0%) in 2014–2021, and further to 57.3% (95% CI: 42.3–71.6%) in 2021–2025. However, the differences among the time periods were not statistically significant (*p* = 0.085). Geographically stratified analysis indicated no significant differences across regions (*p* = 0.152). The Northwest region exhibited the highest weighted pooled prevalence at 61.8% (95% CI: 45.6–75.7%), while the Central-east and Northeast regions showed relatively lower rates at 43.6% (95% CI: 30.4–57.8%) and 42.8% (95% CI: 24.8–63.1%), respectively. The North and South regions presented intermediate prevalence rates at 54.9% (95% CI: 34.7–73.5%) and 46.2% (95% CI: 20.8–73.8%), respectively (Figure 3); data for the Tibet region were missing and thus excluded from the analysis.

Subgroup analysis by production type (layers, broilers, and breeders) showed that the pooled prevalence of MS was 46.0% (95% CI: 29.2–63.3%) in layers, 49.4% (95% CI: 38.6–60.3%) in broilers, and 69.6% (95% CI: 40.5–92.0%) in breeder chickens. Although no statistically significant difference was observed among production types (*p* = 0.137), breeder flocks still exhibited a relatively higher prevalence of MS. Age-based subgroup analysis demonstrated a statistically significant difference between age groups (*p* < 0.001). The pooled prevalence of MS was 35.2% (95% CI: 22.7–59.9%) in chickens younger than 90 days and 40.6% (95% CI: 11.9–63.2%) in chickens older than 90 days.

Based on molecular detection methods, the pooled prevalence of MS showed only slight variation across different periods, ranging from 21.7% (95% CI: 7.4–40.8%) during 2014–2021 to 22.2% (95% CI: 9.1–39.1%) during 2021–2025. Although no further increase was observed in the later period, the difference between periods was not statistically significant (*p* = 0.427), suggesting that the overall prevalence of MS has remained relatively stable in recent years. No significant differences were observed among seasonal subgroups (*p* = 0.181). Spring showed the highest weighted pooled prevalence at 53.0% (95% CI: 42.3–63.5%), followed by summer at 33.6% (95% CI: 13.7–66.0%), while autumn and winter exhibited lower prevalence rates at 34.6% (95% CI: 36.4–43.3%) and 38.5% (95% CI: 12.3–68.9%), respectively.

### 3.5. Publication Bias and Sensitivity Analysis Results

**Figure 4:**
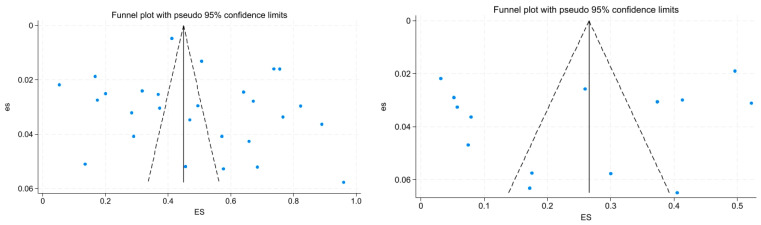
Funnel plots of MS prevalence (**left:** serological detection; **right:** molecular detection).

**Figure 5:**
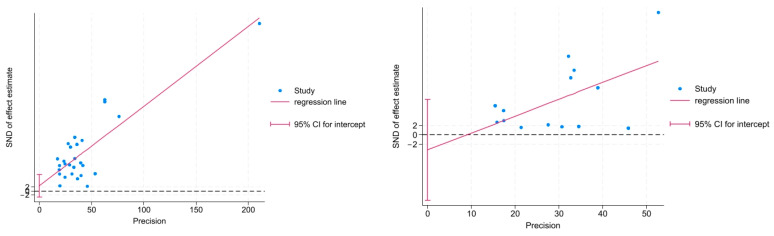
Egger’s test for the prevalence of MS (**left:** serological detection; **right**: molecular detection).

Publication bias was evaluated using funnel plots and Egger’s regression test. The funnel plot displayed a mild asymmetry in the distribution of the included studies, suggesting the potential presence of some degree of publication bias (Figure 4). However, the subsequent Egger’s test results demonstrated that neither the serological detection (*p* = 0.354) nor the molecular detection (*p* = 0.536) reached statistical significance (both *p* > 0.05), indicating no significant publication bias (Figure 5). Given that Egger’s test yielded no evidence of substantial publication bias and the asymmetry in the funnel plot was minor, the trim-and-fill method was not further employed to adjust the pooled effect size [49].

**Figure 6:**
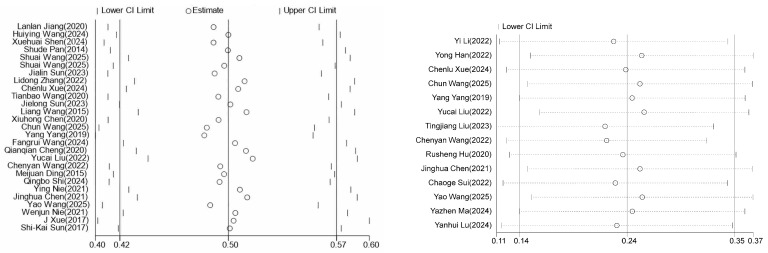
Sensitivity analysis of MS prevalence based on different detection methods (**left:** serological assays; **right:** molecular detection-based methods) [1,8,18,19,20,21,22,23,24,25,26,27,28,29,30,31,32,33,34,35,36,37,38,39,40,41,42,43,44,45,46,47,48].

Sensitivity analysis was performed using the leave-one-out method, where individual studies were sequentially excluded. The results indicated that for serological detection, the overall pooled estimate shifted from the baseline of 0.50, ranging narrowly between 0.48 and 0.53. The 95% confidence intervals exhibited minimal variation and remained tightly clustered and stable, demonstrating the high robustness of the findings (Figure 6, left panel). Conversely, for molecular detection, the effect sizes showed slightly larger fluctuations due to the relatively small number of included studies (Figure 6, right panel); nevertheless, this did not alter the overall conclusions.

## 4. Discussion

Through systematic review and meta-analysis, this study provides the first comprehensive quantitative synthesis of the infection status of MS in chicken flocks in Mainland China. Compared with previous localized or fragmented reports, this study incorporates a more extensive dataset from recent years, thereby increasing the overall sample size and geographical coverage to provide the most comprehensive evidence-based assessment currently available for MS infection in mainland China. MS infection is characterized by chronic respiratory and systemic colonization with intermittent shedding, allowing infected flocks to remain long-term reservoirs for both horizontal and vertical transmission. The overall subgroup analysis demonstrated that the pooled antibody seroprevalence estimated via serological detection (49.7%, 95% CI: 41.4–58.1%) was extremely significantly higher than the pooled nucleic acid/molecular positivity rate estimated via molecular detection (21.9%, 95% CI: 11.9–34.0%), with a highly significant statistical difference observed between the two diagnostic typologies (*p* < 0.001). This pronounced discrepancy highlights the fundamental epidemiological divergence between antibody persistence and active infection. Seropositivity not only reflects field strain exposure but is also heavily confounded by historical infections or residual maternal/vaccine-induced antibodies. Conversely, molecular detection directly targets nucleic acid-positive samples, serving as a more sensitive and rigorous indicator of active shedding and ongoing transmission within flocks [50]. Maternal antibodies may influence serological interpretation in young chicks; however, their protective effect against MS colonization and transmission remains limited and controversial. The statistical divergence between serological and nucleic acid detection prevalence is primarily governed by their distinct diagnostic principles and the biological characteristics of their respective “window periods.” Serological methods (e.g., ELISA, serum plate agglutination [SPA], and hemagglutination inhibition [HI]) detect host humoral immune products. Once generated, these antibodies can persist for weeks, months, or even throughout the entire production cycle of the flock [51]. In contrast, molecular detection relies heavily on nucleic acid amplification, which is strictly restricted to the narrow window of active pathogen replication and shedding. If flocks enter an intermittent shedding phase or if viral/bacterial load is suppressed under immune pressure, nucleic acid testing can easily yield false-negative results. Furthermore, the widespread implementation of commercial live (e.g., MS-H strain) or inactivated MS vaccines in Chinese layer and breeder farms in recent years introduces substantial interference. Conventional serological tools lack the differentiating-infected-from-vaccinated-animals (DIVA) capability, thereby inevitably inflating the pooled seroprevalence [52]. Nucleic acid detection assays, conversely, are minimally affected by inactivated vaccines, making them far more objective in mapping the true baseline burden of field-strain transmission. Therefore, when interpreting epidemiological data, it is imperative to rigorously differentiate the clinical implications of these two testing strategies rather than misinterpreting high seroprevalence as immediate infection pressure. The stratified pooling strategy implemented in this meta-analysis was specifically designed to eliminate effect-size bias triggered by such methodological heterogeneity. From a global perspective, leading poultry-producing nations face parallel challenges in MS eradication. For instance, Spain reported a commercial layer farm seroprevalence ranging from 30.7% to 81.2% [6], and Croatia documented a seroprevalence exceeding 60% in commercial flocks [7], underscoring that MS remains a recalcitrant pathogen exerting a continuous global threat to the poultry industry. While advanced poultry-producing regions like the United States and several European countries have successfully limited MS infection to negligible levels through rigorous, long-term eradication programs in great-grandparent and grandparent flocks [53], our findings indicate a distinct epidemiological situation in mainland China. The estimated pooled seroprevalence was substantially higher than the pooled molecular detection prevalence, highlighting important differences between the serological and molecular surveillance results. However, epidemiological data were unavailable for several regions, including the Tibet Autonomous Region. Therefore, the pooled estimates should be interpreted as reflecting currently available evidence rather than a fully representative nationwide prevalence profile. Collectively, these findings underscore the need for continued surveillance and improved understanding of MS epidemiology to support the development of evidence-based control strategies.

Regarding temporal distribution, the serological subgroup analysis tracked an upward trajectory from 38.2% in 2010–2014 to 57.3% in 2021–2025. Although the absolute numerical increase warrants attention, the available data do not support a definitive temporal trend. This shifting trend may be associated with the structural transformation of China’s poultry industry over the past decade, characterized by a rapid transition from small-scale farming to high-density, intensive operations. High stocking densities, multi-age rearing systems, and incomplete eradication of vertical transmission chains in breeder sectors may be related to the continued circulation of MS within biosecure-compromised facilities [1,9]. Additionally, an advanced industry-wide awareness regarding MS-induced pathologies (e.g., infectious synovitis, egg production drops, and eggshell apical abnormalities) has accelerated the deployment of highly sensitive commercial ELISA kits, which partly explains the elevated detection rates in later studies. Nonetheless, because the primary literature synthesized herein exhibited massive fluctuations in sampling randomness, breed composition, and complex vaccination backgrounds, and given the sparse literature density in the early period (only 4 studies in 2010–2014), a definitive nationwide linear growth model cannot be conclusively deduced. Future multicenter, cross-regional prospective cohort studies executing standardized national surveillance protocols are urgently required to validate the true spatiotemporal dynamics of MS.

Age-specific subgroup analysis revealed statistically significant differences across age cohorts (*p* < 0.001). The weighted pooled MS prevalence in chickens older than 90 days of age (40.6%) was significantly higher than that in chickens younger than 90 days of age (35.2%). This dynamic indicates that an extended rearing cycle significantly escalates the cumulative risk of environmental exposure and horizontal transmission among individual birds. These insights establish a precise, data-driven foundation for deciphering the spatiotemporal heterogeneity and age-dependent susceptibility of MS in China, thereby facilitating the design of tailored, region-specific control measures.

Geographically, the Northwest region exhibited the highest serological pooled prevalence (61.8%), whereas relatively lower rates were observed in Central-east and Northeast China. However, the inter-regional differences were not statistically significant (*p* = 0.152), and therefore the following interpretations should be regarded as potential explanations rather than definitive causes. This spatial disparity may be associated with variations in breeding stock introduction frequencies, farming modalities, and the stringency of biosecurity enforcement. As a major destination for white-feathered broiler and layer introduction in China, the Northwest region may face an elevated risk of vertical transmission from parental stock. Furthermore, the arid climate coupled with drastic diurnal temperature fluctuations in this region could contribute to respiratory stress in poultry, thereby increasing their susceptibility to pathogens. This vulnerability may be further influenced by limited laboratory diagnostic coverage at the grassroots level in certain areas, which may impede the timely identification and culling of infected flocks, leading to a cumulative increase in antibody-positive cases. Conversely, despite its massive production volume, Central-east China maintains a lower serological prevalence; this may be related to a higher degree of industrialization, more robust corporate self-testing and official surveillance systems, and a stronger collective awareness regarding MS eradication. Notably, these inter-regional differences did not attain statistical significance (*p* = 0.152), and the number of included studies and sampling timeframes varied across regions. These limitations suggest that current data are insufficient to support a rigorous regional risk stratification, underscoring the urgent need for future comprehensive cross-sectional surveys that encompass all provinces under a unified sampling framework.

No statistically significant difference in MS prevalence was observed among different production types (layers, broilers, and breeders; *p* = 0.137), suggesting that this pathogen poses a widespread and ubiquitous threat across diverse poultry production systems in China. Notably, the breeder population maintained a relatively high pooled prevalence of 69.6%; however, because differences among production types were not statistically significant, this finding should be interpreted as an observed epidemiological trend rather than conclusive evidence of increased infection risk. This relatively high prevalence may be associated with prolonged rearing periods, multi-age management practices, and the persistent nature of vertical transmission. Concurrently, accumulating evidence suggests that persistent infection and shedding in breeder flocks may contribute to pathogen maintenance and transmission within poultry production systems, potentially affecting the health status of progeny offspring [6]. Furthermore, the extremely wide 95% confidence interval (CI: 40.5–92.0%) delineated for the breeder cohort reflects a profound inter-study heterogeneity. This pronounced variability might result from uneven biosecurity standards and the disparate MS eradication levels implemented across different breeding facilities. Although the differences among production types did not reach statistical significance, breeder flocks consistently exhibited the highest pooled prevalence. This finding is epidemiologically important because breeder populations represent the primary upstream source of vertical transmission within integrated poultry production systems. Compared with commercial layer flocks, breeder and broiler production systems generally experience greater economic pressure associated with MS infection, including reduced hatchability, impaired growth performance, infectious synovitis, and persistent transmission through parental stocks. Consequently, MS surveillance and eradication programs have historically focused more heavily on breeder and broiler industries than on the layer sector. The relatively high prevalence observed in breeder flocks in the present study further supports the importance of strengthening MS monitoring and eradication strategies at the breeder level. Notably, MS-associated infectious synovitis has been increasingly reported in native chicken breeding systems in China, suggesting that clinical manifestations may vary among production systems and host populations [1]. However, insufficient reporting in the primary studies prevented further quantitative assessment in the present analysis.

Despite these surveillance and eradication efforts, complete elimination of MS remains challenging in many production systems. In commercial poultry production, antimicrobial administration remains one of the most commonly adopted approaches for controlling clinical MS-associated disease, particularly in breeder and broiler systems. However, long-term or repeated antibiotic usage may suppress pathogen loads without eliminating chronic colonization, thereby complicating epidemiological interpretation and potentially masking active transmission. More importantly, excessive antimicrobial use may contribute to the emergence of antimicrobial-resistant Mycoplasma strains, antimicrobial residues, and broader One Health concerns associated with antimicrobial resistance (AMR) dissemination across animal–human-environment interfaces [54]. Therefore, future MS control strategies should increasingly emphasize integrated approaches combining breeder eradication, biosecurity optimization, molecular surveillance, vaccination management, and prudent antimicrobial stewardship rather than relying primarily on antibiotic intervention.

An additional source of heterogeneity may arise from the grouping of multiple serological assays and nucleic acid-based detection methods into broad diagnostic categories. Different serological methods, including ELISA, serum plate agglutination (SPA), and hemagglutination inhibition (HI), exhibit substantial variation in sensitivity, specificity, and susceptibility to nonspecific reactions. Moreover, serological positivity may not necessarily indicate active infection because antibody responses can persist long after exposure or vaccination. Transient false-positive serological reactions may also occur following certain vaccination programs or immune stimulation, potentially contributing to overestimation of pooled seroprevalence. In addition, vaccination history may further complicate interpretation of serological results. Inactivated MS bacterins can induce strong antibody responses detectable by SPA and ELISA but do not necessarily prevent persistent colonization or shedding, whereas the MS-H live vaccine may produce variable serological responses under field conditions. Therefore, serological positivity may represent a mixture of field exposure, vaccine-induced antibody responses, and chronic colonization rather than active infection alone.

Similarly, molecular detection methods differ in analytical sensitivity, sampling requirements, and their ability to detect intermittent shedding. Detection rates may also vary according to sample type, such as respiratory swabs, air sacs, synovial tissues, or other clinical specimens, which may differ in pathogen load and diagnostic sensitivity. Therefore, the pooled estimates presented in this study should be interpreted within the context of methodological heterogeneity among diagnostic assays and sampling strategies.

Furthermore, molecular epidemiological approaches can facilitate differentiation between vaccine-associated detections and field infections, thereby improving the interpretation of surveillance data. However, recent evidence suggests that vlhA-based genotyping has important limitations for epidemiological tracing because the vlhA gene is subject to strong host immune selection pressure and may undergo substantial sequence variation during persistent colonization [55]. Consequently, vlhA typing alone may not reliably differentiate vaccine-derived strains from circulating field strains. In addition, the coexistence of MS-H vaccine strains and wild-type strains within vaccinated flocks does not necessarily indicate vaccination failure, but may instead reflect the complex ecology of persistent colonization and mixed-strain circulation in commercial poultry systems [56]. Recent advances in molecular diagnostics, including validated PCR-based DIVA assays and commercially available diagnostic kits capable of differentiating MS-H vaccine strains from field isolates, have improved the accuracy of MS surveillance and epidemiological investigations. These tools provide valuable support for disease monitoring and control programs. In addition, although the MS-H vaccine has demonstrated good efficacy in controlling MS infection, occasional studies have reported genetic changes in vaccine-derived strains under field conditions. Therefore, continuous molecular surveillance remains important to monitor potential alterations in strain characteristics and to distinguish vaccine-derived strains from circulating field isolates. Furthermore, interpretation of molecular results should be integrated with vaccination history, clinical findings, and epidemiological information to accurately assess infection dynamics within poultry populations.

The hierarchical structure of modern poultry breeding systems may also influence MS transmission dynamics. In vertically integrated production systems, infection occurring in grandparent or parent breeder stocks may facilitate downstream dissemination through vertical transmission to commercial production flocks. However, insufficient reporting detail in the original studies prevented further stratified analysis according to breeding hierarchy. At present, no unified nationwide governmental eradication program specifically targeting MS has been implemented in China. Control measures are primarily managed by individual poultry enterprises, particularly within breeder and integrated broiler production systems where the economic impact of vertical transmission and chronic infection is more substantial. Consequently, surveillance intensity and biosecurity standards may vary considerably among different production sectors and geographic regions.

The extremely high heterogeneity observed in the present meta-analysis (I^2^ > 99%) suggests substantial variability among the included studies in terms of geographic region, production system, flock management, sampling strategy, diagnostic methodology, and vaccination background. Therefore, the pooled prevalence estimates should be interpreted cautiously and regarded primarily as overall reference estimates rather than precise representations of a uniform national prevalence level.

The absence of widely applied diagnostic tools complicates the interpretation of serological surveillance data, as positive results may reflect field exposure, vaccination-induced antibody responses, or persistent colonization. On this basis, serological prevalence estimates should not be interpreted solely as indicators of active infection pressure. Vaccination background may represent an important but largely unmeasured source of heterogeneity in the present study. Because most original studies did not report vaccine type, vaccination history, or immunization schedules, further meta-regression analysis was precluded. For this reason, part of the observed serological prevalence may reflect vaccine-induced antibody responses rather than natural exposure alone.

## 5. Limitations

Several limitations should be considered. First, substantial heterogeneity remained among studies despite the use of random-effects models and subgroup analyses, likely reflecting differences in geographic regions, production systems, management practices, diagnostic methods, and vaccination backgrounds. Therefore, the pooled estimates should be interpreted as overall reference values rather than precise national prevalence indicators. Second, serological and molecular assays differ in their diagnostic performance and biological interpretation. In particular, serological positivity may reflect natural exposure, chronic colonization, or vaccination-induced antibody responses. Because vaccination histories were incompletely reported, the potential influence of bacterin- and MS-H-associated serological responses could not be evaluated. Third, epidemiological data were unavailable for some regions, and most studies originated from commercial poultry systems. In addition, information on clinical status, MS-associated disease outcomes, strain characteristics, and chicken breeds was often lacking. Consequently, the representativeness of the findings for underrepresented areas and backyard flocks was limited, and potential differences among flock types and chicken populations could not be assessed. Finally, several included studies were derived from Chinese-language postgraduate theses, and publication bias cannot be completely excluded, suggesting that studies with low prevalence or negative findings may have been underrepresented. Consequently, the results should be interpreted with caution.

## 6. Conclusions

This meta-analysis suggests that Mycoplasma synoviae (MS) is widely present in chicken populations in mainland China. However, substantial between-study heterogeneity suggests that pooled prevalence estimates should be interpreted as overall reference values rather than precise national prevalence indicators. Differences between serological and molecular detection results may reflect variation in diagnostic methods, vaccination background, infection dynamics, and sampling strategies. Although prevalence estimates varied across regions, production types, and age groups, these findings should be interpreted cautiously because not all subgroup differences were statistically significant. Future studies incorporating standardized surveillance protocols, comprehensive vaccination information, and longitudinal data are needed to further clarify the epidemiology of MS and support evidence-based control strategies.

## Figures and Tables

**Table 1:** Baseline characteristics of the included studies.

Author	Sample Year	Sample Area	MS-Ser/Total	Proportion	MS- NA/Total	Proportion
Pan et al., 2014 [18]	2010	Northeast	2940/5800	50.69%	N/A	N/A
Wang, 2015 [19]	2012–2014	Central-east	476/2849	16.71%	N/A	N/A
Sun et al., 2017 [1]	2010–2015	Multi-regional	389/829	46.92%	N/A	N/A
Xue et al., 2017 [8]	2010–2015	Multi-regional	18,285/44,395	41.19%	N/A	N/A
Ding et al., 2015 [20]	2015	Central-east	207/359	57.66%	N/A	N/A
Chen et al., 2020 [21]	2018	Northwest	866/1289	67.18%	N/A	N/A
Wang et al., 2020 [22]	2018–2020	Southwest	252/368	68.48%	N/A	N/A
Jiang et al., 2020 [23]	2019	North	2956/3910	75.60%	N/A	N/A
Nie et al., 2021 [24]	2019–2020	Northwest	275/969	28.38%	N/A	N/A
Cheng, 2020 [25]	2020	North	231/1325	17.43%	N/A	N/A
Nie et al., 2021 [26]	2020	Central-east	573/1555	36.85%	N/A	N/A
Zhang, 2022 [27]	2020–2021	Northeast	318/1588	20.03%	N/A	N/A
Sun et al., 2023 [28]	2020–2021	Northwest	2887/3914	73.76%	N/A	N/A
Shen et al., 2024 [29]	2022	Central-east	676/882	76.64%	N/A	N/A
Wang(1), 2025 [30]	2022	Northeast	174/600	29.00%	N/A	N/A
Wang(2), 2025 [30]	2022	Northeast	343/600	57.17%	N/A	N/A
Sun et al., 2023 [31]	2022–2023	Central-east	169/371	45.55%	N/A	N/A
Wang, 2024 [32]	2022–2023	Southwest	568/1148	49.48%	N/A	N/A
Wang et al., 2024 [33]	2023	North	403/1081	37.28%	N/A	N/A
Shi et al., 2024 [34]	2024	North	362/550	65.82%	N/A	N/A
Hu, 2020 [35]	2018–2020	Central-east	N/A	N/A	90/300	30.00%
Han, 2022 [36]	2019–2021	Central-east	N/A	N/A	54/940	5.74%
Sui, 2022 [37]	2019–2021	Central-east	N/A	N/A	96/237	40.51%
Li, 2022 [38]	2020–2021	South	N/A	N/A	462/1118	46.92%
Liu, 2023 [39]	2022–2023	Central-east	N/A	N/A	540/1034	52.22%
Ma et al., 2024 [40]	2022–2023	Northwest	N/A	N/A	43/250	17.20%
Lu et al., 2024 [41]	2023	Northwest	N/A	N/A	399/1068	37.36%
Liu et al., 2022 [42]	2017–2021	Central-east	111/2100	5.29%	66/2100	3.14%
Yang et al., 2019 [43]	2018	South	288/300	96.00%	53/302	17.55%
Wang et al., 2022 [44]	2018–2020	Central-east	1069/1668	64.09%	1378/2777	49.62%
Chen et al., 2021 [45]	2021	Central-east	52/384	13.54%	34/455	7.47%
Xue, 2024 [46]	2022–2023	South	548/1726	31.75%	392/1510	2.58%
Wang et al., 2025 [47]	2023	Central-east	938/1140	82.28%	62/1190	5.21%
Wang, 2025 [48]	2023–2024	Central-east	674/757	89.04%	60/757	7.93%

Note: MS, *Mycoplasma synoviae*; Ser, seroprevalence-positive count; NA, nucleic acid-prevalence-positive count; Total, total number of inspected individuals; N/A, not available; Multi-regional, 21 provinces.

**Table 2:** Subgroup comparisons of MS prevalence across different diagnostic systems.

Variable	Number of Positive Cases	Sample Size	Pooled Prevalence		I^2^	*p*
			ES	95% CI		
Molecular Detection	3729	14,038 *	0.219	0.119–0.340	99.61	0.000
Serological Detection	37,030	82,457 *	0.497	0.414–0.581	99.87	

* ES: effect size; Among the included studies, some employed both nucleic acid and serological detection simultaneously.

**Table 3:** Subgroup Analysis Based on Serological and Molecular Detection.

Variable	Number of Positive Cases	Sample Size	Pooled Prevalence		I^2^	*p*
			ES	95% CI		
Serological Detection						
Sampling Year						
2010–2014	22,090	53,873	0.382	0.264–0.507	99.72	0.085
2014–2021	10,085	19,729	0.476	0.304–0.650	99.84	
2021–2025	4855	8855	0.573	0.423–0.716	99.51	
Sampling Region						
Northeast	3775	8588	0.428	0.24–0.631	99.53	0.152
North	3590	6316	0.549	0.347–0.735	99.89	
Central-east	5493	13,791	0.436	0.304–0.578	99.82	
South	1108	1816	0.462	0.208–0.738	99.40	
Northwest	4390	6722	0.618	0.456–0.757	99.57	
Production Type						
Layers	6575	11,316	0.460	0.292–0.633	99.70	0.137
Broilers	4574	9202	0.494	0.386–0.603	98.65	
Breeders	2784	5906	0.696	0.405–0.920	99.74	
Age						
<90 Days	599	2664	0.352	0.119–0.632	99.38	0.000
≥90 Days	758	2012	0.406	0.227–0.599	98.24	
Molecular Detection						
Sampling Year						
2014–2021	2233	8229	0.217	0.074–0.408	99.70	0.427
2021–2025	1496	5809	0.222	0.091–0.391	99.50	
Season						
Spring	281	537	0.530	0.423–0.635	82.89	0.181
Summer	506	1137	0.336	0.137–0.660	99.65	
Autumn	347	970	0.346	0.264–0.433	86.59	
Winter	366	1048	0.385	0.123–0.689	98.85	

## Data Availability

The data presented in this study were derived from published articles available in the public domain. All extracted data values, study characteristics, and quality assessments are provided in Table 1. The original sources are listed in the References Section and are accessible via their respective DOIs.

## Supplementary Materials

The following supporting information can be downloaded at https://www.mdpi.com/article/10.3390/ani16121893/s1. Data S1: production type; Data S2: Detailed domain-specific scores and final quality tier assignments for each study.
